# Supplementary material for: DDX5 Functions as a Tumor Suppressor in Tongue Cancer
Source: Cancers (Basel). 2023 Dec 18;15(24):5882. doi: 10.3390/cancers15245882 (PMC10741615; doi:10.3390/cancers15245882)
Supplement: Supplementary file 1 [file cancers-15-05882-s001.zip › cancers-2706732-supplementary.pdf]

Supplementary materials

DDX5 Functions as a Tumor Suppressor in Tongue Cancer

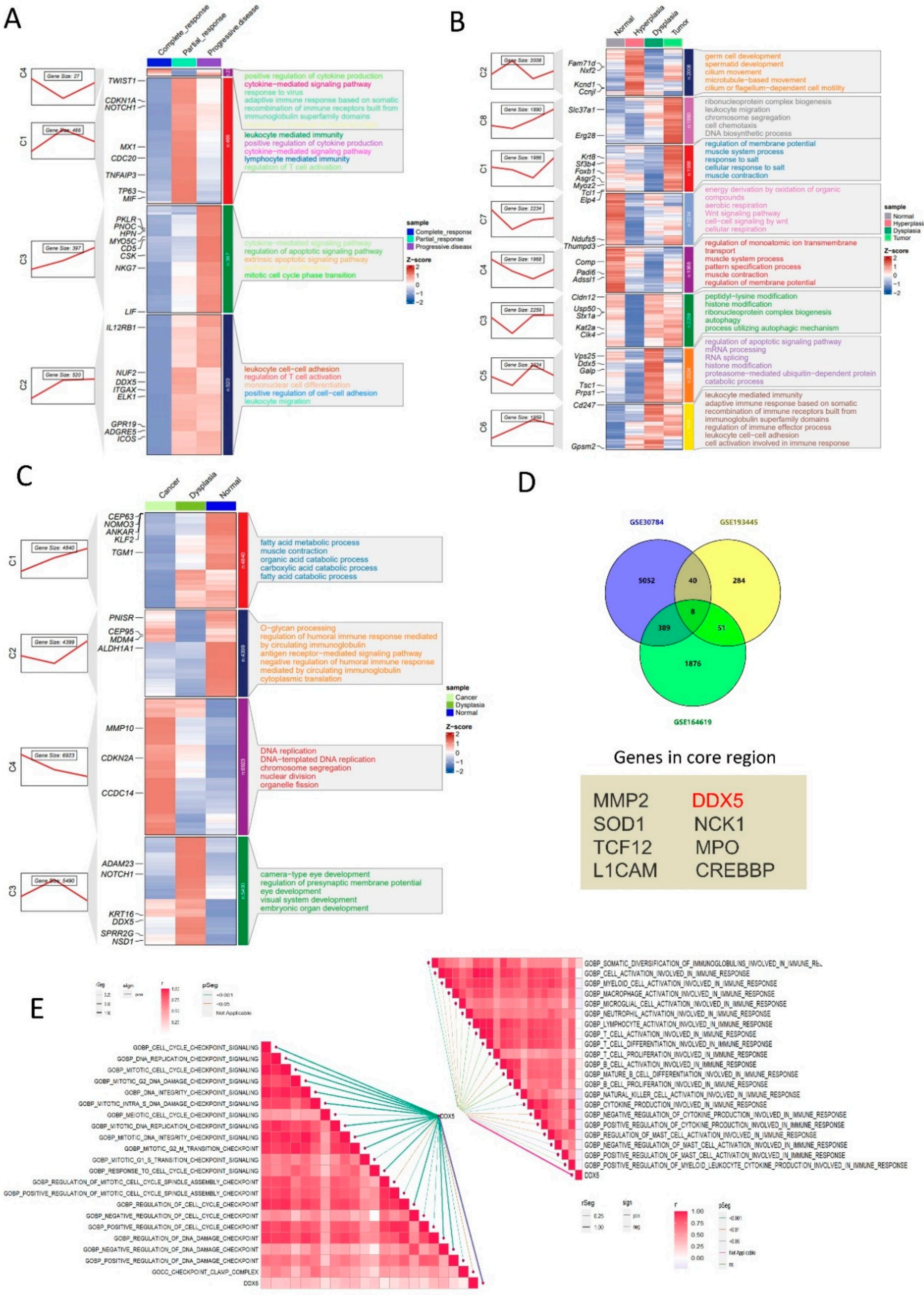

Figure S1 DDX5 expression is positively associated with OS of tongue cancer patients.

(A) The series of diagrams illustrate the patterns of dynamic changes in DEGs during the treatment of tongue cancer using ClusterGVis (R package) from GSE193445.

(B) The series of diagrams illustrate the patterns of dynamic changes in DEGs during the progression of tongue cancer using ClusterGVis (R package) from GSE164619.

(C) The series of diagrams illustrate the patterns of dynamic changes in DEGs during the progression of tongue cancer using ClusterGVis (R package) from GSE30784.

(D) Venn diagram showing the overlap of tongue cancer related genes identified in this study from three datasets: GSE193445, GSE164619, GSE30784

(E) Correlations between DDX5 and the enrichment scores of immunotherapy-predicted pathways

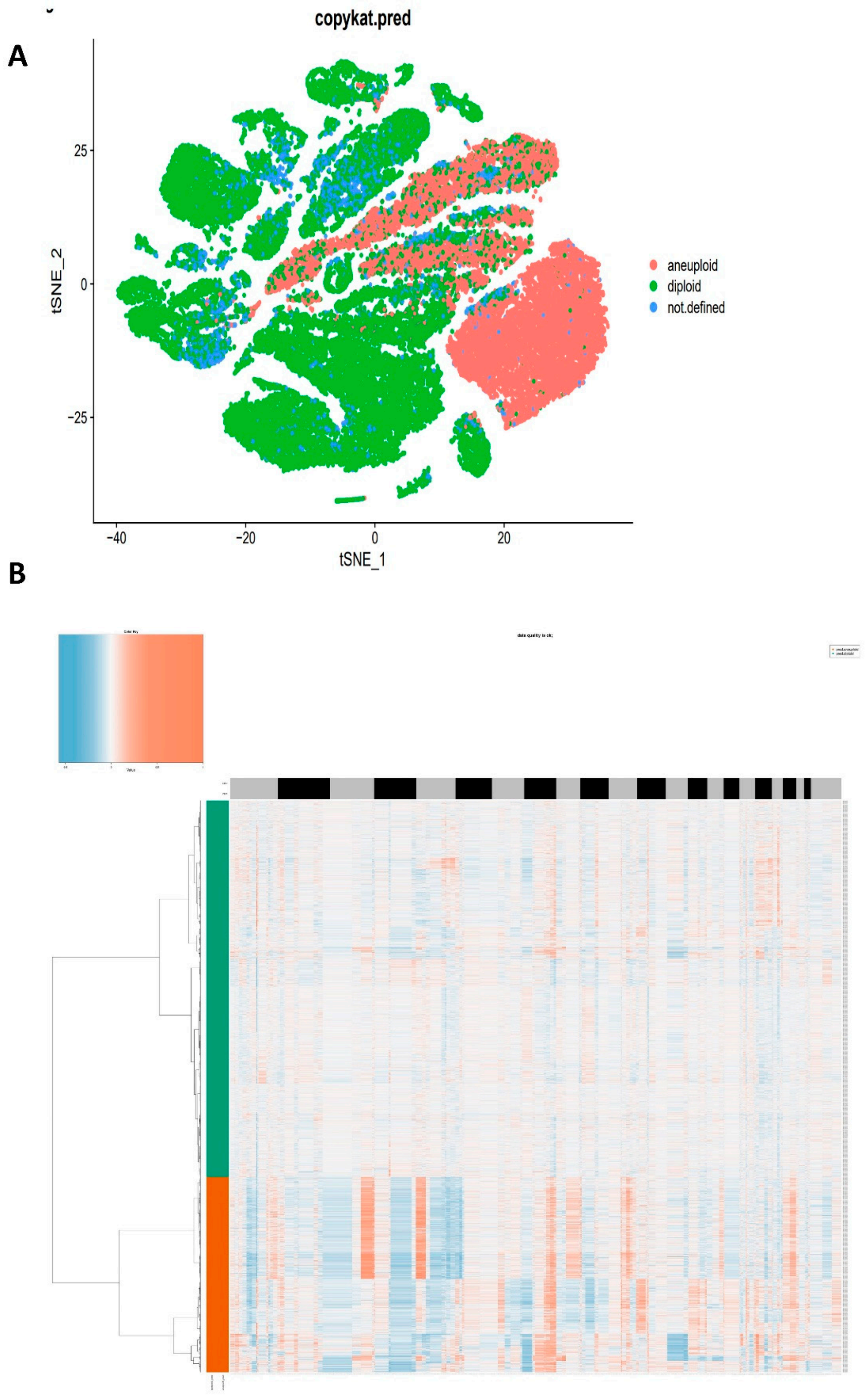

Figure S2 Single cell analysis using CopyKAT

(A) The t-SNE diagrams of tumor and normal cells in tongue cancer tissues are represented by different colors.

(B) Clustered heat maps for single cell copy number profiles estimated by CopyKAT. Green represents normal cells and orange represents malignant cells, judged by CopyKAT.

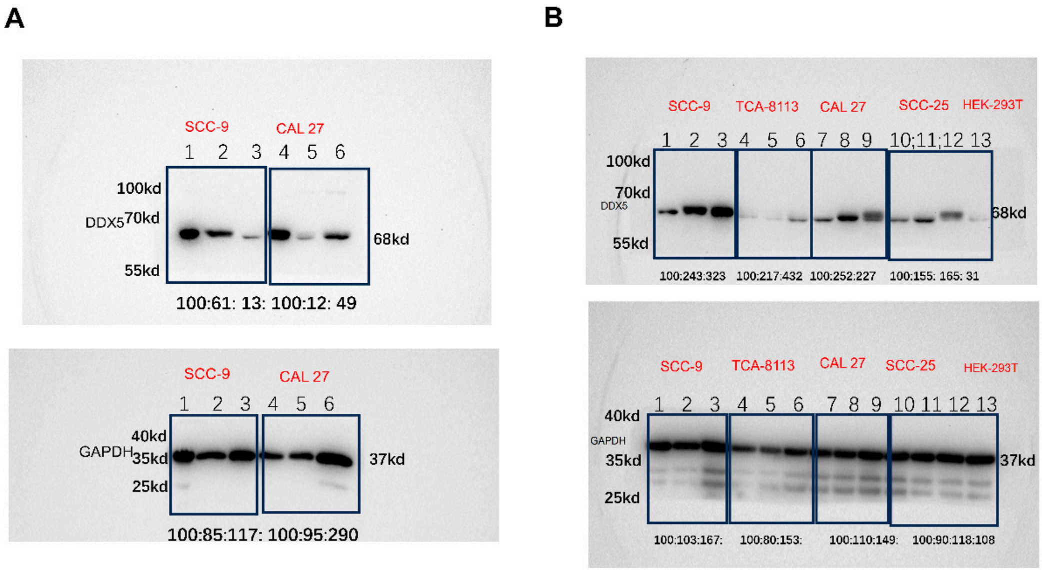

Figure S3 Western blot

(A) Knockdown efficiency in DDX5-KD and control counterpart tongue cancer cells (Figure 1D). include densitometry readings/intensity ratio of each band

(B) Overexpression efficiency in DDX5-OE and control counterpart tongue cancer cells (Figure 2A).
